# Arbuscular Mycorrhizal Fungi and Their Relationships with the Soil Nutrients and Heavy Metals in Ancient Trees in Blue-Crowned Laughingthrush Habitats

**DOI:** 10.3390/jof11110776

**Published:** 2025-10-28

**Authors:** Hui Li, Pei Wei, Kongzhong Xiao, Wei Liu, Weiwei Zhang

**Affiliations:** 1 National Conservafon and Rescarch Conter for Biue-Crowned Laughingthrush, Jiangxi Provincial Key Laboratory of Conservation Biology, Jiangxi Agricultural University, Nanchang 330045, China; 2022994834@nit.edu.cn (H.L.); m18679635046@163.com (P.W.); 2 College of Forestry, Jiangxi Agricultural University, Nanchang 330045, China; 3 School of Civil and Architectural Engineering, Jiangxi University of Water Resources and Electric Power, No. 289 of Tianxiang Avenue, Nanchang 330099, China; 4 School of Soil and Water Conservation, Jiangxi University of Water Resources and Electric Power, No. 289 of Tianxiang Avenue, Nanchang 330099, China; 2022994828@nit.edu.cn

**Keywords:** ancient trees, heavy metal contamination, Shuikoulin forest, arbuscular mycorrhizal fungi, rhizosphere soil

## Abstract

The fragile ancient ‘Shuikoulin’ forests, which provide critical habitats for the critically endangered Blue-crowned Laughingthrush, are increasingly degraded by soil contamination and heavy metal pollution. This study examines the rhizosphere environment of four key ancient tree species in the bird’s core habitat, focusing on soil properties, heavy metal accumulation, and the structure of arbuscular mycorrhizal (AM) fungal communities. The results revealed that *Liquidambar formosana* showed the highest total nitrogen (TN) and available phosphorus (AP), whereas *Quercus chenii* had the lowest soil organic matter (SOM). The primary heavy metal contaminant across all tree species was Cd (*Igeo* > 2), followed by the metalloid As. We detected 41 AM fungal species spanning 7 genera, with *Glomus* dominating (84.19% relative abundance). OTU richness was highest in *Cinnamomum camphora* and *L. formosana* (110 each), followed by *Q. chenii* (88) and *Castanopsis sclerophylla* (75). Structural equation modeling indicated that soil nutrients (TN, TP, AP, SOM) suppressed the accumulation of V, Cr, Ni, and Cu, thereby indirectly favoring *Glomus* and *Paraglomus*. In contrast, higher pH and total potassium (TK) levels promoted Co and Zn bioavailability and negatively affected *Acaulospora* and other minor genera. Tree species identity directly modulated these interactions. Our findings demonstrate that ancient tree species shape AM fungal assembly through distinct rhizosphere geochemical niches, providing a mechanistic basis for restoring degraded habitats critical to endangered species conservation.

## 1. Introduction

The blue-crowned laughingthrush (*Garrulax courtoisi*) is a critically endangered passerine bird endemic to China, with a global population of approximately 250 mature individuals [1,2]. The survival of this species is intrinsically linked to the integrity of ancient “Shuikoulin” forest communities, which are dominated by trees such as *Cinnamomum camphora* (*C. camphora*), *Liquidambar formosana* (*L. formosana*), *Castanopsis sclerophylla* (*C. sclerophylla*), and *Quercus chenii* (*Q. chenii*) [3,4]. These forests provide the essential structural and ecological basis for the bird’s breeding and survival [3,5]. Consequently, the health of the ancient tree communities directly influences the population dynamics of the blue-crowned laughingthrush [5,6].

Ancient trees represent integral and ecologically significant components of forest ecosystems, possessing substantial ecological and cultural value. Their vitality serves as a critical indicator of soil health, yet in recent years, the escalating burden of soil pollution, particularly from heavy metals such as arsenic (As) and cadmium (Cd), along with nutrient imbalances, has severely threatened their survival. These pollutants disrupt soil microbial communities, inhibit enzymatic activity, and induce oxidative stress, thereby compromising nutrient uptake and physiological functions in ancient trees [7,8]. Within the soil microbiome, arbuscular mycorrhizal (AM) fungi form critical symbiotic relationships with plant roots, enhancing nutrient uptake, mitigating heavy metal stress, and contributing to overall ecosystem resilience [9,10]. While soil bacteria also play significant roles in heavy metal biogeochemistry, this study specifically focuses on AM fungi for several compelling reasons. The perennial nature and deep root systems of these ancient trees suggest a long-term, stable relationship with AM fungi, which may be disproportionately important for their nutrient acquisition and stress tolerance in a contaminated and degrading soil environment [11]. The direct physical connection formed by AM fungi between the soil and the root system positions them as first responders to soil conditions [12], potentially making their community composition a more direct indicator of tree health in the context of the studied stressors [13]. Therefore, elucidating the interactions among ancient trees, soil properties (including heavy metals), and AM fungal communities is a critical first step.

Current research on this interplay suffers from three major limitations: (i) an oversimplification of tree species differences, wherein studies focus on single species and neglect community-level diversity in laughingthrush habitats, leaving patterns of AM fungal divergence among key tree rhizospheres poorly characterized; (ii) unclear mechanistic drivers, specifically how soil nutrient attributes regulate heavy metal bioavailability and thereby shape AM fungal community assembly; and (iii) a disconnect from practical application, as conventional conservation measures (e.g., revetment repair) often fail to incorporate microbial remediation strategies, potentially delaying ecosystem recovery. To address these gaps, this study aims to characterize the physicochemical properties, heavy metal contamination profiles, and AM fungal communities in the rhizosphere soils of key ancient tree species, and to identify the primary edaphic factors governing AM fungal community structure. The findings will provide a scientific foundation for updating the conservation action plan for the blue-crowned laughingthrush and promote the integration of microbiome-based strategies into ecological restoration standards.

## 2. Materials and Methods

### 2.1. Study Area

**Figure 1:**
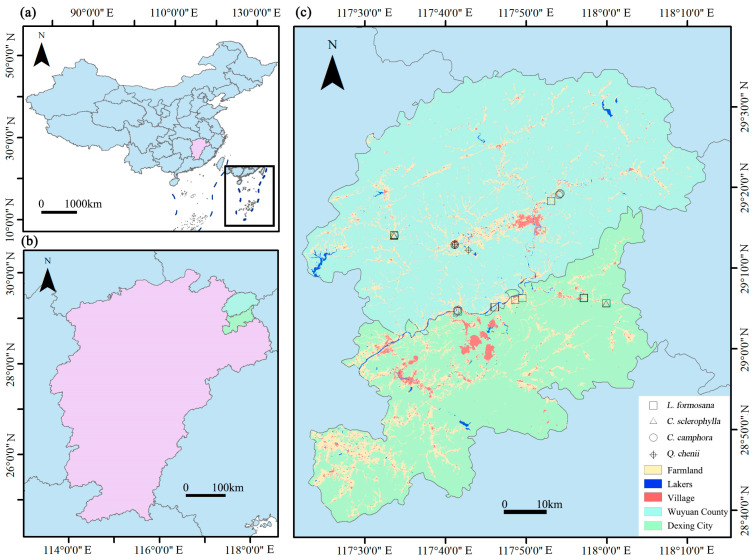
Location of the study area (**a**,**b**) and the 34 sampling sites (**c**).

The research was conducted in Wuyuan County and Dexing City, Jiangxi Province, China (117°13′17.76″–118°11′21.48″ E, 28°44′17.16″–29°38′38.04″ N) (Figure 1a,b), a region characterized by a subtropical humid monsoon climate with distinct seasonal variations and high annual precipitation. This area constitutes the core habitat of the critically endangered blue-crowned laughingthrush. Although famous for its pristine landscapes, the growth of ancient trees within this habitat has been compromised by anthropogenic disturbances and natural stressors, as documented in recent ecological assessments [3].

### 2.2. Soil Sample Collection

Soil samples were collected between 17 and 19 October 2022, from eight nesting sites of the blue-crowned laughingthrush, targeting four ancient tree species (*C. camphora*, *L. formosana*, *C. sclerophylla*, and *Q. chenii*) known to have nesting histories (Figure 1c). Three to four replicates were taken per species. Within the zones of dense fine roots beneath the tree drip line, soil cores with a diameter of 3 cm were extracted from four cardinal directions at depths of 0–20 cm and 20–40 cm. Samples from each tree were homogenized after removing roots, litter, and debris, resulting in 34 composite samples. All sampling tools were thoroughly sterilized with 75% ethanol between collecting samples from different trees to prevent cross-contamination. Subsamples were stored at −80 °C for MiSeq sequencing and at −20 °C for soil chemistry and heavy metal quantification.

### 2.3. Soil Chemical Property Analysis

Soil chemical properties were analyzed following standardized protocols [14]. Air-dried samples were processed as follows: pH was measured using a composite electrode (at a soil:water ratio of 1:2.5 *w*/*v*; total nitrogen (TN) was determined via a continuous flow analyzer; total potassium (TK) was measured by flame photometry; total phosphorus (TP) was assessed through H_2_SO_4_-HClO_4_ digestion with molybdenum–antimony colorimetry; available phosphorus (AP) was extracted using NaHCO_3_ followed by molybdenum–antimony colorimetry; and soil organic matter (SOM) was quantified using the K_2_Cr_2_O_7_ external heating method.

### 2.4. Soil AM Fungal High-Throughput Sequencing

Genomic DNA was extracted from 5 g soil samples and amplified using AM fungal-specific primers AMV4.5NF (5′-AAGCTCGTAGTTGAATTTCG-3′) and AMDGR (5′-CCCCAACTATCCTCATTAATCAT-3′). Preliminary experiments determined the minimum number of PCR cycles, with three replicates per sample; PCR products were pooled, verified via 2% agarose gel electrophoresis, and purified using an AxyPrep DNA Gel Extraction Kit (Sigma-Aldrich (Shanghai) Trading Co., Ltd., Shanghai, China). The purified DNA was eluted in Tris-HCl buffer, quantified using the QuantiFluor™-ST system, mixed proportionally, denatured with sodium hydroxide (NaOH), and sequenced on an Illumina HiSeq250 platform. Paired-end (PE) reads were assembled via overlap, quality-controlled, filtered, and clustered into operational taxonomic units (OTUs) at 97% similarity. Representative sequences were taxonomically classified by blasting (BLASTn 2.5.0+) against the MaarjAM database (https://www.maarjam.botany.ut.ee, accessed on 10 January 2023) [15].

### 2.5. Soil Heavy Metal Analysis and Contamination Assessment

Heavy metal concentrations, including vanadium (V), chromium (Cr), cobalt (Co), nickel (Ni), copper (Cu), zinc (Zn), arsenic (As) and cadmium (Cd), were quantified using microwave digestion in conjunction with inductively coupled plasma mass spectrometry (ICP-MS). The microwave digestion-ICP-MS method determined the total concentrations of the heavy metals, encompassing both bioavailable (labile) and non-labile (insoluble) forms.

The geoaccumulation index (*Igeo*) was applied to assess the contamination from heavy metals, calculated using the formula:

*Igeo* = *log*_2_ (Cn/(1.5 × Bn) (1)

where Cn (mg/kg) is the measured metal concentration, Bn (mg/kg) denotes the geochemical background values for Jiangxi Province [16], and the factor of 1.5 accounts for lithogenic variability in trace metal concentrations.

### 2.6. Data Analysis

Geographic maps were generated using ArcGIS 10.8 and Photoshop 2019. Statistical analyses were conducted in SPSS 25, where one-way ANOVA with LSD post hoc tests were used to compare variations in soil chemistry, AM fungal communities, and heavy metal concentrations across samples (with significance thresholds set at *p* < 0.05 for significant differences and *p* < 0.01 for highly significant differences). Structural equation modeling (SEM) was employed to quantify relationships among AM fungal taxa (at the genus level), soil properties, and heavy metals. Data preprocessing involved boxplot-based outlier detection (with outliers replaced by group means) and normality assessment through scatterplots: Pearson correlation was applied for normally distributed data, while Spearman rank correlation was used for non-normal data. Visualizations were created using Origin 2021 (for bar charts), Canoco 5 (for redundancy analysis), and R software (v.3.5.2, https://www.r-project.org/) (for clustering/correlation heatmaps).

## 3. Results

### 3.1. Rhizosphere Soil Physicochemical Properties of Ancient Tree Species

Significant interspecific variations were observed in the soil physicochemical properties of the rhizosphere of the studied ancient tree species (Table 1). *L. formosana* exhibited the highest TN and AP concentrations, which were significantly greater than those of *Q. chenii*. TN and AP levels under *C. camphora* and *C. sclerophylla* were intermediate and not significantly different from those under *L. formosana* or *Q. chenii*. SOM concentration under *Q. chenii* (6.39 ± 1.90 g·kg^−1^) was significantly lower than under *C. camphora* (21.20 ± 4.22 g·kg^−1^), *L. formosana* (23.08 ± 1.82 g·kg^−1^), and *C. sclerophylla* (28.74 ± 3.11 g·kg^−1^), which showed similar SOM values.

### 3.2. Soil Heavy Metal Characteristics in Rhizosphere Soils of Ancient Tree Species

The distribution of heavy metal content in the rhizosphere soil varied considerably across the different ancient tree species, with significant differences observed primarily among the essential trace elements (Table 2). Regarding the essential trace elements, *C. camphora* and *Q. chenii* generally exhibited higher accumulation levels. The content of V and Cr was significantly higher in the soil of *Q. chenii* compared to that of *L. formosana* (*p* < 0.05). In contrast, the Zn content under *Q. chenii* was significantly lower than that under *C. camphora*. *C. camphora* showed the highest mean concentrations of Co, Ni, Cu, and Zn among all species. For the non-essential toxic elements, Arsenic As and Cd, no statistically significant differences were found among the tree species. However, it is noteworthy that the mean As content under *C. camphora* was substantially higher than that under the other species. All species exhibited similarly low levels of Cd contamination. In summary, the results indicate that tree species identity is a significant factor influencing the distribution, and potentially the bio-accumulation, of heavy metals in the rhizosphere, particularly for essential trace elements.

**Figure 2:**
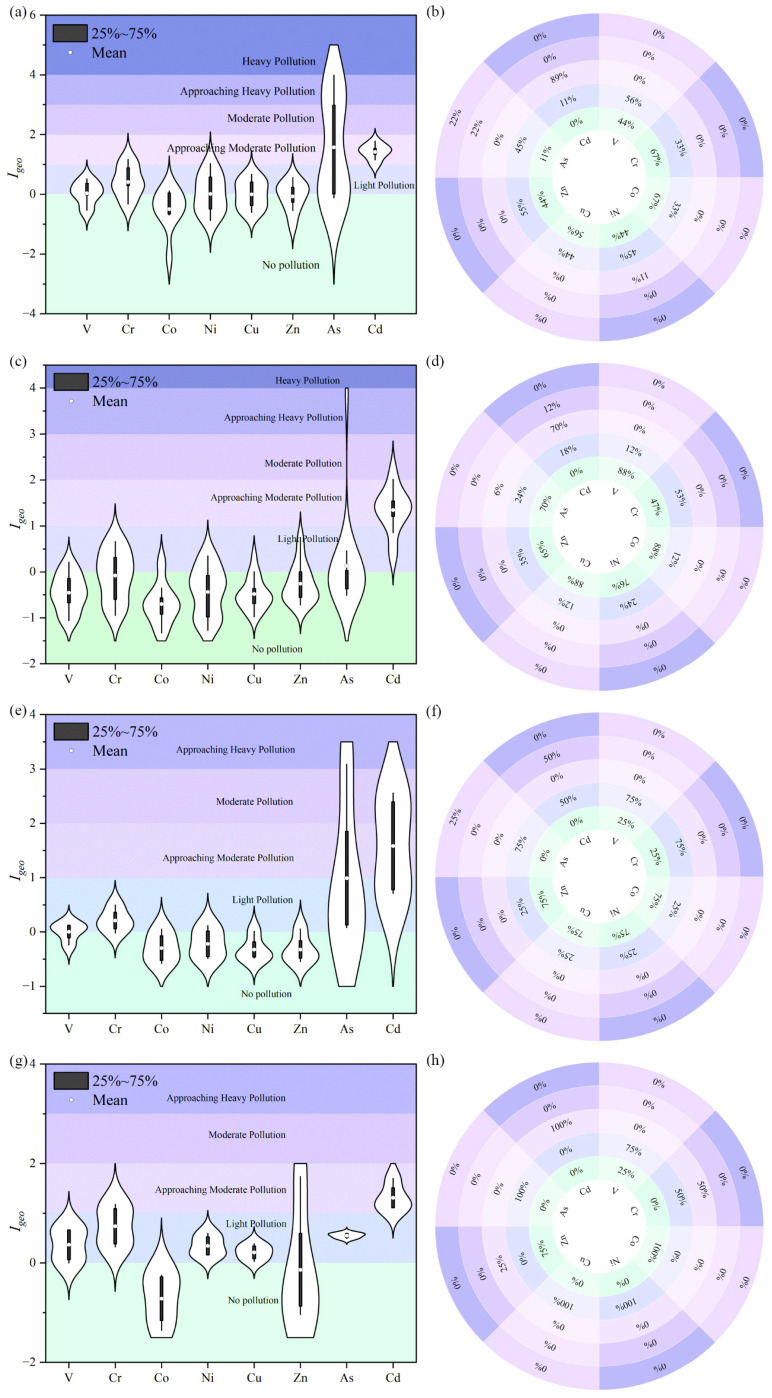
The assessment results of the geocumulative index (*Igeo*) for soil heavy metal content and pollution levels, distributed among the roots of various ancient trees. Panels (**a**,**b**) correspond to *C. camphora*; panels (**c**,**d**) to *L. formosana*; panels (**e**,**f**) to *C. sclerophylla*; and panels (**g**,**h**) to *Q. chenii*.

Soil heavy metal contamination was assessed using the geochemical background values of Jiangxi Province. The pollution levels were categorized according to the geoaccumulation index (*Igeo*) as follows: *Igeo* < 0 (no pollution), 0–1 (light pollution), 1–2 (approaching moderate pollution), 2–3 (moderate pollution), 3–4 (approaching heavy pollution), and >4 (heavy pollution). The quantification results of *Igeo* for the rhizosphere soil of *C. camphora* are presented in Figure 2a. As and Cd exhibit moderate to heavy pollution, whereas the other six heavy metal elements show slight or no pollution. Figure 2b illustrates that As has the most varied pollution levels in *C. camphora*, with 11%, 45%, 22%, and 22% of samples exhibiting no, slight, moderate, and moderate to heavy pollution, respectively. The quantification results of *Igeo* in the rhizosphere soil of *L. formosana* are shown in Figure 2c. Except for Cd, which exhibits moderate to heavy pollution, and As, which shows slight pollution, the *Igeo* values of the other six heavy metals indicate no pollution. Figure 2d indicates that Cd appears in 18%, 71%, and 12% of *L. formosana* samples as slight, moderate to heavy, and moderate pollution, respectively. The quantification results of *Igeo* in the rhizosphere soil of *C. sclerophylla* are shown in Figure 2e, where Cd is the most severely polluted element, while the other elements exhibit slight pollution or no pollution. Figure 2f reveals that As appears in 75% and 25% of *C. sclerophylla* samples as slight and moderate to heavy pollution, respectively, and Cd appears in 50% of samples as both slight and moderate pollution. The quantification results of *Igeo* in the rhizosphere soil of *Q. chenii* are shown in Figure 2g, with Cd being the most severely polluted element, while the other elements exhibit slight pollution or no pollution. Figure 2h indicates that the *Igeo* values of all eight elements are less than 2, with Cd showing moderate to heavy pollution in all *Q. chenii* samples, and Cr appearing in 50% of samples as both slight and moderate to heavy pollution.

### 3.3. Rhizosphere Soil Fungal Community Structure

**Figure 3:**
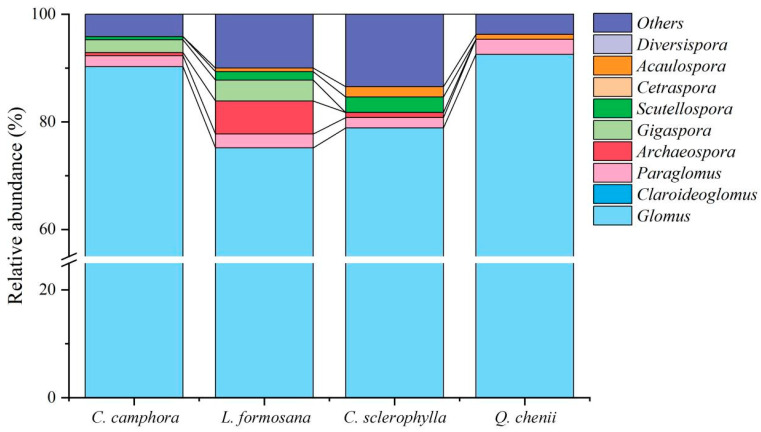
Community composition of soil arbuscular mycorrhizal (AM) fungi in the inter-root zone of various ancient trees (at the genus level).

High-throughput sequencing of 34 rhizosphere soil samples from four tree species yielded 680,181 sequences, with an average of 20,005 valid sequences per sample. *C. sclerophylla* exhibited the highest average number of valid sequences (32,632), while sequence lengths varied from 213 to 220 base pairs. Clustering at 97% similarity identified 402 operational taxonomic units (OTUs), which were refined to 114 after filtering. Taxonomic analysis revealed 41 AM fungal species across 7 genera, 7 families, and 5 orders. In total, the genus *Glomus* was dominant, accounting for 84.19% of the total sequences (Figure 3). Compared with *C. camphora*, *L. formosana*, and *C. sclerophylla*, the relative abundance of *Glomus* in *Q. chenii* was the highest. *L. formosana* contained the largest number of AM fungal genera, whereas *Q. chenii* had the fewest, with only four genera identified: *Glomus*, *Paraglomus*, *Acaulospora*, and *Others*.

**Figure 4:**
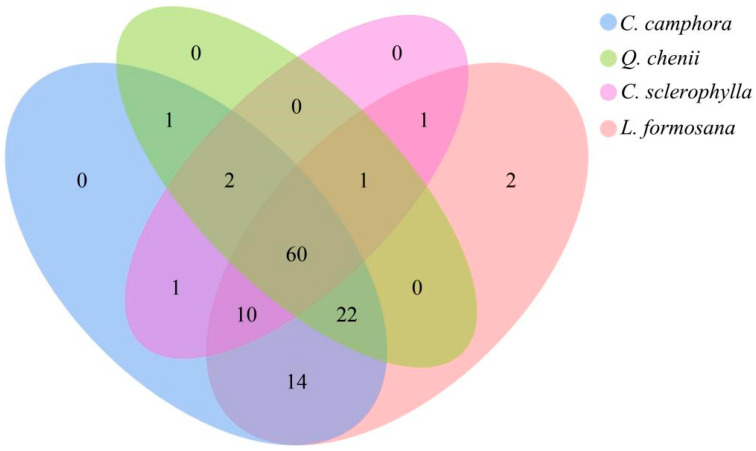
Horizontal distribution map of AM fungal genera in the inter-root soils of various ancient trees.

A total of 383 OTUs were obtained from the rhizosphere soil of the four ancient tree species at a 97% similarity threshold (Figure 4). The number of AM fungal OTUs in each species’ rhizosphere soil was as follows: *C. camphora* (110), *L. formosana* (110), *Q. chenii* (88), and *C. sclerophylla* (75). Only *L. formosana* possessed unique OTUs, numbering 2. No unique OTUs were detected in the rhizosphere soil of the other three tree species. The highest number of shared OTUs (107 OTUs, accounting for 27.94% of the total OTUs) was observed between *C. camphora* and *L. formosana*. In contrast, the fewest shared OTUs (63 OTUs, accounting for 16.45% of the total OTUs) occurred between *Q. chenii* and *C. sclerophylla*. These results indicate a greater similarity in AM fungal community composition between *C. camphora* and *L. formosana*, while *Q. chenii* and *C. sclerophylla* exhibited more distinct AM fungal communities.

### 3.4. Correlation Analysis Between Soil Environmental Factors and AM Fungal Community Composition

**Figure 5:**
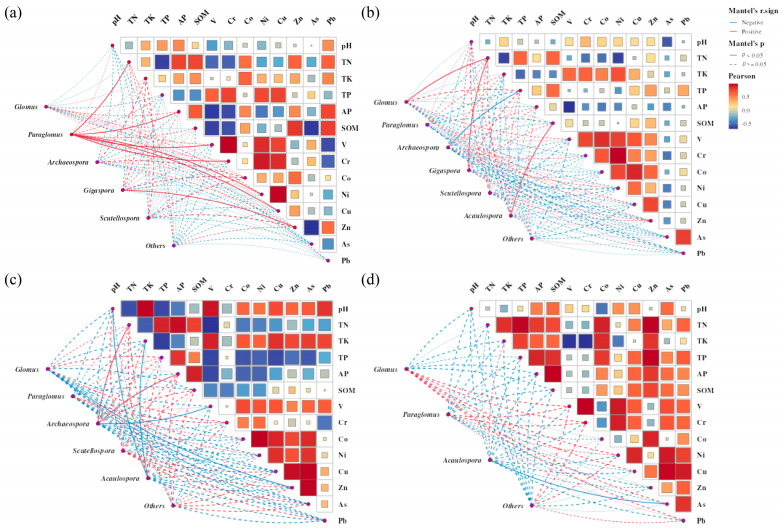
The correlation analysis between soil environmental factors and the distribution of AM fungal communities at the genus level for *C. camphora* (**a**), *L. formosana* (**b**), *C. sclerophylla* (**c**) and *Q. chenii* (**d**). Mantel’s *p* and Mantel’s r values represent the significance level (*p*-value) and the correlation coefficient statistic (r), respectively. Line colors and styles indicate statistical significance: red lines denote positive correlations, blue lines indicate negative correlations, solid lines signify statistically significant relationships (*p* < 0.05) between the matrix and connected variables, whereas dashed lines denote non-significant relationships (*p* ≥ 0.05).

Mantel tests were employed to identify key factors influencing the genus-level composition of AM fungi in the rhizosphere soil of four ancient tree species by correlating community structure with soil physicochemical properties and heavy metal contents (Figure 5). In the rhizosphere soil of *C. camphora* (Figure 5a), the relative abundance of *Glomus* exhibited significant positive correlations with TN, AP, V, Cr, Ni, and Cu contents, while *Gigaspora* showed a significant positive correlation with Zn content. For *L. formosana* (Figure 5b), the relative abundances of both *Glomus* and *Gigaspora* were significantly positively correlated only with TN content; *Acaulospora* demonstrated a significant positive correlation with SOM content, whereas *Archaeospora* exhibited a significant negative correlation with TP content. In the rhizosphere soil of *C. sclerophylla* (Figure 5c), the relative abundance of *Archaeospora* was significantly positively correlated with TN and AP contents but significantly negatively correlated with V content; *Glomus* showed a significant negative correlation with Zn content, and *Acaulospora* was significantly negatively correlated with soil pH and TK content. In both *L. formosana* and *C. sclerophylla*, a statistically significant negative correlation was observed between TN and TK concentrations (*p* < 0.05). Conversely, in *Q. chenii* (Figure 5d) rhizosphere soil, only *Acaulospora* displayed a significant negative correlation, specifically with As content.

### 3.5. Structural Equation Modeling of AM Fungal Community Distribution and Soil Environmental Factors

**Figure 6:**
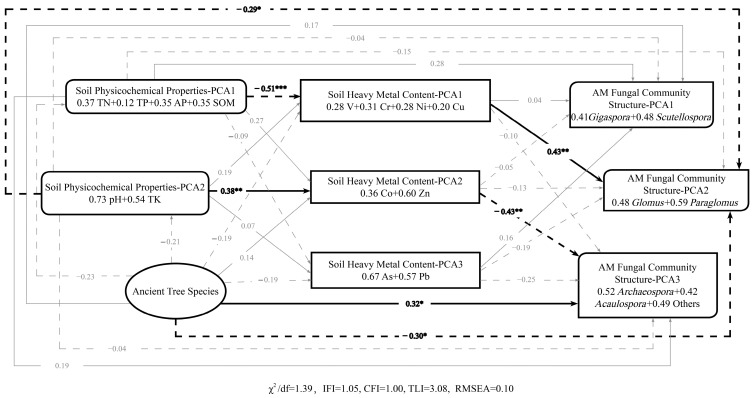
Structural equation modeling of inter-root soil microbes and environmental factors in different ancient trees. Standardized values were used. Black arrows represent significant impacts, while gray arrows represent non-significant impacts. Solid arrows indicate positive effects, whereas dashed arrows indicate negative effects. *** indicates *p* < 0.001, ** indicates *p* < 0.01, and * indicates *p* < 0.05.

Utilizing principal component analysis (PCA) and structural equation modeling (SEM), we clarified the interactions between AM fungal communities and soil environmental factors (refer to Figure 6). Elevated levels of TN, TP, AP, and SOM were found to suppress the accumulation of V, Cr, Ni, and Cu, thereby indirectly promoting the abundance of *Glomus* and *Paraglomus* AM fungi (mediation effect: −0.51 × 0.43 = −0.22). At the same time, increased pH and TK levels facilitated the accumulation of Co and Zn, indirectly decreasing the colonization by *Acaulospora* and other genera (mediation effect: 0.38 × −0.43 = −0.16). Moreover, higher pH and TK levels directly inhibited the proliferation of *Glomus* and *Paraglomus*. Additionally, the ancient tree species exerted direct regulatory effects on the abundance of AM fungi from the genera *Glomus*, *Paraglomus*, *Acaulospora*, and *Others*.

## 4. Discussion

### 4.1. Characteristics and Ecological Implications of Rhizosphere Nutrient Cycling in Ancient Trees

This study reveals significant differences in rhizosphere soil nutrient characteristics among different ancient tree species. *L. formosana* exhibited the highest nitrogen and phosphorus availability (TN: 1.92 g·kg^−1^; AP: 0.82 mg·kg^−1^), while *C. sclerophylla* accumulated the highest level of soil organic matter (SOM: 28.74 g·kg^−1^). These differences primarily stem from the functional traits of tree species: recent studies indicate that deciduous trees significantly alter rhizosphere microbial community structure through litter input and root exudates, thereby influencing organic matter decomposition and nutrient cycling processes [17,18]. As a deciduous species, the high-quality litter input of *L. formosana* promotes nutrient cycling and creates “nutrient hotspots,” providing optimal growing conditions for understory vegetation and food sources for birds. In contrast, the tannin-rich litter of *Q. chenii* delays the decomposition process by inhibiting hydrolase activity [19], resulting in reduced nutrient availability. This nutrient heterogeneity directly affects the spatial structure and resource distribution of the habitat, significantly influencing the foraging behavior and nest site selection of the blue-crowned laughingthrush [5]. Notably, the microhabitat heterogeneity created by ancient trees through rhizosphere processes may be a key mechanism for maintaining habitat biodiversity [20,21,22].

### 4.2. Heavy Metal Contamination Patterns and Ecological Risks

The widespread cadmium contamination (*Igeo* > 2) in the study area poses a serious ecological threat, with *Q. chenii* rhizosphere showing the highest Cd content. This high Cd concentration can be transferred and biomagnified through the soil-invertebrate-bird food chain. Research has proven that even environmentally relevant concentrations of Cd exposure can affect avian reproductive success and immune function [23,24,25], posing a direct threat to the survival of the blue-crowned laughingthrush populations.

Different tree species exhibited distinct heavy metal enrichment characteristics: the significant enrichment of vanadium and chromium in *Q. chenii* and the specific accumulation of Zn in *C. camphora* may reflect their evolutionarily adapted rhizosphere filtration mechanisms [26]. Latest molecular ecology research has found that this element-specific enrichment capacity is closely related to specifically expressed metal transporter genes in roots [27,28]. Of particular concern is the As contamination in *C. camphora* rhizosphere (33% of samples showed moderate to severe pollution), which may affect bird health through groundwater pathways. Microbiome studies reveal that the expression levels of As metabolism genes in rhizosphere microorganisms directly regulate arsenic speciation and bioavailability [29,30].

### 4.3. AM Fungal Communities and Ecosystem Resilience

This study is the first to systematically characterize the AM fungal communities in the rhizosphere of ancient trees in the blue-crowned laughingthrush habitats. *C. camphora* and *L. formosana* supported higher AM fungal diversity (110 OTUs), while *Q. chenii* showed the lowest fungal diversity (88 OTUs). This difference was significantly positively correlated with soil organic matter content, validating the “soil organic matter-microbial diversity” coupling hypothesis [31]. AM fungi significantly enhance nutrient acquisition efficiency and stress resistance in trees by extending the host absorption network, serving as crucial symbiotic partners for maintaining tree health [32,33,34].

However, the absolute dominance of *Glomus* (84.19%) indicates simplified community structure and reduced functional redundancy. Other studies reveal that the functional diversity of AM fungi is a better predictor of ecosystem stability than species diversity [35,36]. This reduction in functional redundancy may weaken the resilience of ancient trees to climate change, thereby affecting the ecosystem service functions of the entire habitat [37]. Therefore, maintaining high SOM levels is essential for ensuring AM fungal diversity and functional integrity.

### 4.4. Heavy Metal–Microorganism Interactions and Remediation Potential

AM fungi assist ancient trees in resisting heavy metal stress through various molecular mechanisms: transcriptomic studies show that *Glomus* upregulates glomalin synthesis genes and metallothionein genes to chelate multiple metal ions [38], while *Acaulospora* regulates heavy metal transporter expression and forms physical barriers to limit arsenic translocation to plants [39,40]. The elucidation of these molecular mechanisms provides new perspectives for understanding heavy metal tolerance in plant-microbial symbiotic systems.

Structural equation modeling results confirmed that sufficient nutrients (TN, AP, SOM) can indirectly promote beneficial fungal growth by inhibiting heavy metal accumulation, forming a positive feedback loop of “soil health–fungal function–pollution mitigation.” This finding supports the latest “rhizosphere engineering” theory [41], providing a theoretical basis for using tree–fungal symbiotic systems for habitat restoration. Recent field trials demonstrate that inoculating specific AM fungal strains can significantly improve plant tolerance and fixation efficiency for heavy metals [37,42], offering a technical pathway for ecological restoration of zinc-contaminated areas using *C. camphora*-fungal symbionts.

## 5. Conclusions

This study systematically analyzed the rhizosphere soil environmental characteristics of four key ancient tree species in the habitat of the blue-crowned laughingthrush, revealing significant differences among tree species in terms of nutrient cycling, heavy metal accumulation, and the assembly of AM fungal communities. *L. formosana* exhibited higher nitrogen and phosphorus availability, while the rhizosphere soil of *Q. chenii* was most severely contaminated with cadmium. The AM fungal community was overwhelmingly dominated by *Glomus* (84.19% relative abundance), and its composition was closely correlated with soil nutrients, heavy metal bioavailability, and tree species identity. Structural equation modeling demonstrated that soil nutrients indirectly promoted the growth of beneficial fungi by inhibiting heavy metal accumulation, whereas elevated pH and TK levels enhanced the bioavailability of Co and Zn and suppressed non-dominant genera. These findings provide a scientific basis for the restoration of endangered species habitats from the perspective of rhizosphere micro-environment interactions. For future conservation practices, we recommend: (i) establishing priority protected areas focusing on *L. formosana* and *C. camphora*; (ii) strengthening dynamic monitoring of Cd in the rhizosphere of *Q. chenii* and As in that of *C. camphora*; (iii) maintaining soil organic matter content through litter management to support AM fungal diversity; (iv) utilizing tree–fungal symbiotic systems for ecological restoration of heavy metal-contaminated areas; and (v) incorporating rhizosphere health indicators into the habitat assessment system for the blue-crowned laughingthrush. The identified relationships between tree species, soil chemistry, and AM fungal communities provide a scientific basis for developing targeted rhizosphere management strategies, such as the selection of tree species with beneficial mycorrhizal associations for habitat restoration, to support the conservation of associated endangered species.

## Figures and Tables

**Table 1:** Soil physicochemical properties in the rhizosphere of different ancient tree.

Species Classification	pH	TN (g·kg^−1^)	TK (g·kg^−1^)	TP (g·kg^−1^)	AP (mg·kg^−1^)	SOM (g·kg^−1^)
*C. camphora*	6.09 ± 0.23 a	1.48 ± 0.25 ab	7.84 ± 0.40 a	0.60 ± 0.07 a	0.50 ± 0.11 ab	21.20 ± 4.22 a
*L. formosana*	5.68 ± 0.17 a	1.92 ± 0.18 a	7.54 ± 0.45 a	0.69 ± 0.10 a	0.82 ± 0.12 a	23.08 ± 1.82 a
*C. sclerophylla*	5.27 ± 0.34 a	1.30 ± 0.10 ab	8.10 ± 0.97 a	0.52 ± 0.08 a	0.42 ± 0.12 ab	28.74 ± 3.11 a
*Q. chenii*	5.21 ± 0.38 a	0.76 ± 0.05 b	9.43 ± 0.88 a	0.95 ± 0.20 a	0.06 ± 0.03 b	6.39 ± 1.90 b

Note: The data in the tables represent the mean ± standard error, with a total sample size of 34. Different lowercase letters indicate significant differences (*p* < 0.05) in soil chemical properties among different tree species.

**Table 2:** Characteristics of the distribution of heavy metal content (mg·kg−1) in soil between the roots of different ancient trees.

Species Classification	Essential Trace Elements						Non-Essential Toxic Elements	
	V	Cr	Co	Ni	Cu	Zn	As	Cd
*C. camphora*	132.27 ± 10.94 ab	101.83 ± 12.34 ab	11.24 ± 1.33 a	31.52 ± 4.78 a	32.74 ± 3.54 a	103.82 ± 10.66 a	84.57 ± 29.43 a	0.41 ± 0.07 a
*L. formosana*	94.88 ± 6.32 b	72.73 ± 6.37 b	9.44 ± 0.77 a	22.44 ± 1.96 a	23.04 ± 1.49 a	90.84 ± 7.53 ab	27.57 ± 12.62 a	0.40 ± 0.13 a
*C. sclerophylla*	126.22 ± 7.23 ab	84.13 ± 6.57 ab	12.09 ± 1.22 a	24.89 ± 2.5 a	25.21 ± 2.16 a	83.91 ± 8.22 ab	47.79 ± 28.5 a	0.33 ± 0.12 a
*Q. chenii*	153.65 ± 28.57 a	116.74 ± 23.25 a	8.53 ± 1.29 a	32.64 ± 5.92 a	31.78 ± 4.5 a	63.1 ± 4.52 b	19.4 ± 3.22 a	0.3 ± 0.05 a

Note: The data in the tables are presented as mean ± standard error, with a total sample size of 34. Different lowercase letters denote significant differences (*p* < 0.05) in soil chemical properties among different tree species.

## Data Availability

Data are available upon request. Contact the corresponding author.
